# Therapeutic trough mycophenolic acid levels positively correlate with omega-3 fatty acids in leukocytes of children with idiopathic nephrotic syndrome. a pilot study

**DOI:** 10.3389/fphar.2025.1672304

**Published:** 2025-12-18

**Authors:** Stefano Turolo, Alberto Edefonti, Marie-Louise Syren, Anita S. Bellotti, William Morello, Federica Collino, Carlo Agostoni, Giovanni Montini

**Affiliations:** 1 Fondazione IRCCS Ca’ Granda Ospedale Maggiore Policlinico. Pediatric Nephrology, Dialysis and Transplant Unit, Milan, Italy; 2 Dipartimento di Scienze Cliniche e di Comunità, Dipartimento di Eccellenza 2023-2027, University of Milano, Milan, Italy; 3 Laboratory of Translational Research in Paediatric Nephro-Urology, and Paediatric Nephrology, Dialysis and Transplant Unit, Fondazione Ca’ Granda IRCCS Ospedale Maggiore Policlinico, Milan, Italy; 4 Fondazione IRCCS Ca’ Granda Ospedale Maggiore Policlinico, Pediatric Immuno-rheumatology Unit, Milan, Italy

**Keywords:** fatty acids, leukocyte, mycophenolic acid, nephrotic syndrome, omega-3, pediatrics

## Abstract

The pathogenesis of idiopathic nephrotic syndrome (INS) in most pediatric cases involves immune cell dysfunction, mediated, among other factors, by biologically active polyunsaturated fatty acids -dependent compounds in the blood and cell membranes. The immunosuppressive drug mycophenolate mofetil (MMF) is commonly used to maintain remission in patients with steroid-dependent nephrotic syndrome. The aim of this pilot study was to investigate the effect of trough blood mycophenolic acid (MPA) levels on the endoleukocyte fatty acid omega-3 and omega-6 series. Eighteen pediatric subjects with INS were enrolled, including 12 steroid-sensitive and 6 steroid-resistant children. Fatty acid (FA) profile was analyzed by gas-chromatography, and MPA blood level was evaluated by immunometric assay. Among steroid-sensitive patients, a positive correlation was observed between endoleukocyte omega-3 FA (n-3) and trough blood MPA levels, and a negative correlation was found between endoleukocyte omega-6 FA (n-6) and trough blood MPA levels. In addition, a positive correlation was found between endoleukocyte anti-inflammatory DHA and trough blood MPA levels. In contrast, in steroid-resistant subjects no correlation between endoleukocyte n-6/n-3 ratio and trough blood MPA levels was observed. In conclusion, adequate trough MPA drug levels may have contributed to switch the FA pathway to a more favorable endoleukocyte n-6/n-3 ratio and DHA levels, possibly supporting the natural anti-inflammatory activities of n-3 FA. In steroid-resistant NS, despite adequate MPA levels, no promotion of the anti-inflammatory activity of the n-3 series was found.

## Introduction

Idiopathic nephrotic syndrome (INS) is the most common glomerular disease in children, characterized by proteinuria, hypoalbuminemia and edema. It is caused by podocyte damage, which in most cases depends on immunological mechanisms (Colucci et al., 2019; Noone et al., 2018). Among children with INS, the majority respond to corticosteroids (steroid-sensitive, SS) and have a good prognosis, but those with relapsing proteinuria (steroid-dependent, SD) and those resistant to corticosteroid treatment (SR) may require additional immunosuppressive (IS) therapy, including calcineurin inhibitors (CNI) and mycophenolate mofetil (MMF) (Vivarelli et al., 2017). In addition, approximately half of children with SRNS may eventually require dialysis and/or kidney transplantation (Tullus et al., 2018).

T- and B-lymphocyte-mediated immunity appears to play a central pathogenetic role (Bhatia et al., 2018; Liu et al., 2011; Kitsou et al., 2022; Cortvrindt et al., 2017; Col et al., 2022). In particular, immune cells are involved in the secretion of circulating factors (Candelier and Lorenzo, 2020), which damage the glomerular filtration barrier, triggering proteinuria and the clinical manifestations of INS. T and B lymphocytes are the target of IS drugs.

Mycophenolate mofetil is a prodrug of mycophenolic acid (MPA), which inhibits inosine-5′-monophosphate dehydrogenase, selectively depleting guanosine nucleotides in T and B lymphocytes, thus reducing their proliferation. MPA also inhibits the glycosilation and expression of adhesion molecules, and the recruitment of lymphocytes and monocytes (Allison, 2005).

Fatty acids (FA) have been widely shown to influence and modulate both inflammation and innate immunity (Galli and Calder, 2009). Among them, the long chain, polyunsaturated FA (LC-PUFA) are the most relevant functional compounds, generating the pro- and anti-inflammatory mediators eicosanoids and docosanoids, derived from omega-3 (n-3) and omega-6 (n-6) LC-PUFA.

In autoimmune diseases, higher n-3 blood levels are associated with a reduced inflammatory state (Wendell et al., 2014), while n-6 levels, especially those of arachidonic acid, are associated with an active inflammatory response via T-helper 2 (Willemsen, 2016). Both experimental and randomized clinical trials in humans have shown that pharmacological and/or dietary modification of the FA profile may contribute to shifting inflammation toward a more favorable course in immune-related disorders (Thies et al., 2001). However, studies investigating the relationship between MMF and FA profile in INS are still lacking.

Endoleukocyte FA analysis has recently been described as an alternative to blood FA analysis in the context of INS, as it may better reflect the biological mechanisms underlying NS within leukocytes, which are also the target of IS drugs (Turolo et al., 2025).

The aim of this pilot study was to investigate the effect of trough blood MPA levels on the endoleukocyte FA profile in pediatric patients with SD and SR nephrotic syndrome.

## Patients and methods

### Patients

All the patients with INS less than 18 years old who underwent a routine blood test at the Pediatric Nephrology, Dialysis and Transplant Unit of Fondazione Cà Granda IRCCS Ospedale Maggiore Policlinico, Milan, Italy, between 1st January-31 December 2022, were eligible for the study.

Patients were enrolled with prior written parental consent. The study protocol was approved by the local ethics committee with document number 0035199-U and was conducted according to the declaration of Helsinki.

### Definition of INS

Definitions of SS, SD and SR were applied to INS patients according to international guidelines (Kidney Disease, 2012). Idiopathic nephrotic syndrome was defined by the presence of proteinuria >40 mg/m^2^/hr in a 24 h urine collection, or a spot urine protein to creatinine ratio (uPr/uCr) of >2 mg/mg, serum albumin <2.5 g/dL and edema, in the absence of secondary causes of NS. Patients were divided into two groups, according to their response to the initial corticosteroid treatment and their relapse rate: SDNS and SRNS group. This last group included cases with both subsequent response to IS drugs and multi-drug resistant patients.

### Treatment

The standard medication for the treatment of the first episode and relapses of INS was oral prednisone for all patients, administered according to the IPNA guidelines (Trautmann et al., 2023). At the time of sampling, no patients were taking prednisone treatment.

IS drugs were prescribed to SDNS and SRNS patients. The starting dose of MMF was 1,200 mg/m2 body surface area (maximum dose 3,000 mg), divided into two oral doses every 12 h. Subsequent doses were adjusted based on trough MPA blood levels. As per protocol, patients were monitored every 3 months: the dose was modified in order to achieve a trough level between 3 and 7 mcg/L, in agreement with a recent publication by our group (Morello et al., 2025). Some SDNS and SRNS patients also received tacrolimus at the standard dose of 0.1–0.2 mg/kg/day (maximum dose 10 mg) in 2 doses, to achieve trough blood levels of 3–7 ng/mL. SRNS patients were on combined treatment in order to maintain remission, as recommended in recent guidelines (Morello et al., 2025). Patients who were treated with tacrolimus only were excluded from thise study.

Before and during corticosteroid and IS administration, patients received supportive treatment, either with furosemide, spironolactone or hydrochlorothiazide. If necessary, one or more of the following antihypertensive drugs were also prescribed: ACE-inhibitors, beta blockers and calcium channel blockers.

### Nutritional data/adherence to the mediterranean diet

Since the FA profile is influenced by dietary intake, the exogenous FA intake was estimated in all the patients based on the criterion of adherence to the Mediterranean diet, assessed by the validated Kid Med questionnaire (Serra-Majem et al., 2003) completed by the parents. It consists of 16 items on dietary habits, each to be answered with Yes or No. The minimum value of the resulting score is −4 (low adherence), and the maximum value is +12 (high adherence).

### Blood sample collection and leukocytes separation

Three ml of blood were collected and centrifuged with histopaque-1077 (Sigma Aldrich, St. Louis Missouri, United States) to separate leukocytes. Three ml of histopaque-1077 were transferred to a 15 mL centrifuge tube; 2 mL of blood were mixed with 2 mL of physiological saline solution. The diluted blood was carefully layered over the histopaque, without mixing. The tube was centrifuged at 400 *g* at room temperature for 15 min. The first layer of plasma was removed, and leukocyte layer plus histopaque was collected in a new tube. Leukocytes were washed with physiological saline solution by centrifugation at 160 *g* at room temperature.

### Fatty acid analysis

Isolated PBMCs were methylated with 800 μL of HClMeOH 3N (Sigma Aldrich, St Louis Missouri, United States), incubated for 1h at 90 °C and then refrigerated at 4 °C for 10 min. Afterwards, 2 mL of KCl solution (Sigma Aldrich, St Louis Missouri, United States) and 330 μL hexane (Sigma-Aldrich St Louis Missouri, United States) were added. Samples were then vortexed and then centrifuged at 3,000 rpm for 10 min. Finally, the hexane layer (the upper layer) was collected from each vial and transferred into a gas chromatography vial for FA profile evaluation with gas-chromatograph Shimadzu Nexis GC-2030 (Shimadzu, Kyoto Japan) equipped with a 30 m fused silica capillary column FAMEWAX Restek (Restek Bellefonte, Pennsylvania, United States). Gas chromatography results were analyzed using Labsolution software v. 5.97 SP1 (Shimadzu, Kyoto Japan). Individual FA were expressed as a relative percentage of the total considered FA. Only n-3 and n-6 FA groups were considered for this study analysis.

### Biochemical analyses

Urinary protein (uPr), urinary creatinine (UCr), blood white cell count, Fk and MPA trough blood levels (Co), serum triglycerides, total cholesterol and HDL cholesterol were measured and the uPr/uCr ratio was calculated, as part of patients’ routine analyses.

Blood was collected for MPA analysis 12 h after drug administration.

MPA was measured with EMIT II plus assay, with the Dimension X Pand analyzer (Siemens, Germany).

### Statistical analysis

The correlation between FA and MPA data was performed by two tails Spearman bivariate analysis with software SPSS v. 21 (IBM, Armonk, New York, United States). For the analysis, patients were divided into two groups: SDNS patients and SRNS patients.

## Results

Twenty-five children with INS were initially selected for the study. Of them, 18 children (age 11.8 ± 4.9 years, 13 males) treated with MMF, (for a median of 713 days; range 42–4,515),either in combination with tacrolimus, (18 patients) or without it (11 patients), were enrolled in the study. The cohort comprised 12 patients with SDNS and 6 patients with SRNS. Among the SDNS patients, one was in relapse and 11 were in remission at the time of blood sampling. Three out of the 6 SRNS cases were multidrug resistant and had persistent proteinuria.

Demographic, clinical and biochemical data are summarized in Table 1. There were no significant differences between SD and SR patients.

**TABLE 1:** Patients’ demographic, clinical and biochemical data.

Clinical and biochemical data	All patients (18)	SD (12)	SR (6)
Age (years)	11,8 ± 4,9	10,3 ± 3,8	14,0 ± 5,9
UPr/UCr (mg/mg)	0,16 (0,12/0,90)	0,14 (0,12/0,18)	1,09 (0,44/1,59)
Total protein (g/dL)	6,7 (6,22/7)	6,7 (6,5/7)	5,95 (5,22/6,5)
Serum albumin (g/dL)	4,47 (4,21/4,79)	4,5 (4,4/4,79)	3,78 (2,82/4,28)
Triglycerides (mg/dL)	98 (49,5/121,5)	74,5 (50,5/116,5)	101 (46/169)
Cholesterol (mg/dL)	174 (161,5/202,5)	161,5 (147/177,25)	203 (174/211)
HDL chol. (mg/dL)	61,5 (51,5/74,25)	56 (48,5/73,5)	67 (58,5/75)
Patients on MMF + Fk (n)	8	2	6
C0 MPA (ng/mL)	4,05 ± 1,72	4,14 ± 1,66	3,88 ± 1,99

SD, steroid dependent nephrotic syndrome; SR, steroid resistant nephrotic syndrome; UPr, urinary protein; UCr, urinary creatinine; Fk, tacrolimus; MPA, mycophenolic acid; C0, trough blood drug level.Data of Upr/Ucr, Total protein, Serum albumin, Triglycerides and Cholesterol are expresse as median (IQR).

As part of the routine blood tests, all patients had normal liver function, as shown by normal values of transaminases, gamma-glutamyl transferase, cholinesterase and coagulation tests (data not reported).

There was also no difference in the Kid Med score, when comparing patients with SDNS to those with SRNS. As additional information, an average score of 5.03 ± 2.2 (median 7) was observed in the whole population, indicating a suboptimal adherence to the Mediterranean diet.

The n-3 and n-6 endoleukocyte levels in SD and SR patients are summarized in Table 2. There were no significant differences in the FA profile between SD and SR patients.

**TABLE 2:** Endoleukocyte omega-6 and omega-3 levels in SR and SD patients.

Patients id	20:4n6 (AA)	22:6n3 (DHA)	TOT N-3	TOT N-6	N-6/N-3
SR 01	49,24	10,69	15,71	84,23	5,36
SR 02	46,34	8,01	11,89	88,06	7,41
SR 03	37,53	9,45	13,47	86,47	6,42
SR 04	28,23	9,39	14,74	85,2	5,78
SR 05	48,96	5,17	9,309	90,75	9,75
SR 06	40,83	8,11	12,07	87,87	7,28
SD 01	39,97	8,84	14,92	85,02	5,70
SD 02	43,81	6,43	10,45	89,5	8,56
SD 03	49,24	10,69	15,71	84,23	5,36
SD 04	35,01	4,53	8,97	90,99	10,14
SD 05	43,38	7,42	12,16	87,78	7,22
SD 06	47,89	7,52	12,15	87,86	7,23
SD 07	35,64	9,66	15,17	84,79	5,59
SD 08	39,21	3,66	6,07	93,92	15,47
SD 09	46,84	9,97	13,6	86,35	6,35
SD 10	37,54	5,13	8,18	91,77	11,22
SD 11	37,83	3,6	7,83	92,11	11,76
SD 12	41,96	12,1	17,12	82,82	4,84
p-value	n.s	n.s	n.s	n.s	n.s

SR, steroid resistant nephrotic syndrome; SD, steroid dependent nephrotic syndrome. All fatty acids values are expressed as % on the total fatty acids considered.

**FIGURE 1:**
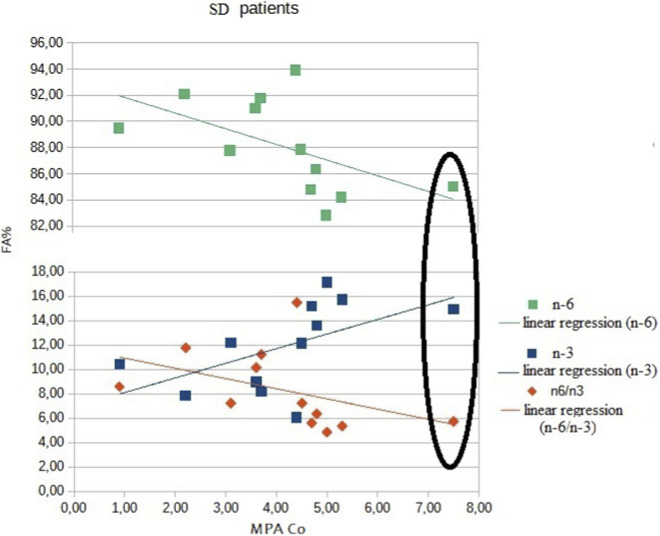
Correlations between trough blood MMF levels and FA profile in SD patients. In steroid-dependent patients, total n-6 fatty acid levels decreased with the increase of MPA trough blood levels, and *vice versa* for n-3 fatty acid levels. As a consequence, the inflammatory index n-6/n-3 ratio, decreased as MPA trough blood levels rised. The only steroid-dependent patient with proteinuria is indicated by a circle. Despite the presence of proteinuria, total n-3, total n-6, and the n-6/n-3 ratio showed the same trend as that of patients without proteinuria.

**FIGURE 2:**
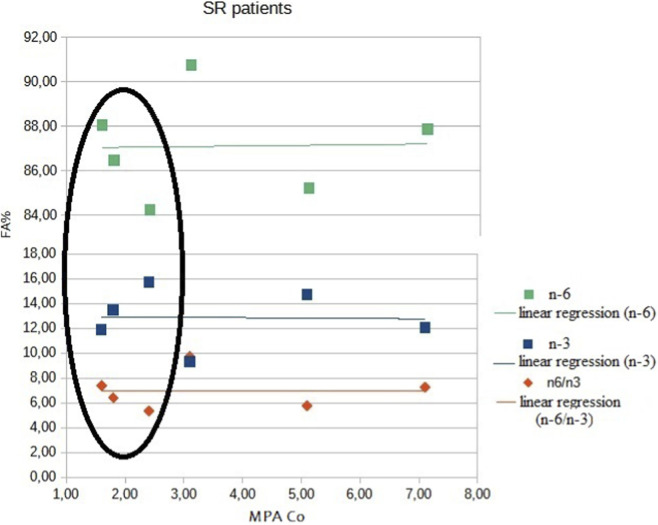
Correlations between trough blood MMF levels and FA profile in SR patients. In steroid-resistant patients, there was no correlation between fatty acids (n-6, n-3, and the n-6/n-3 ratio) and MPA trough blood levels. Despite this, the three patients in remission, indicated by circles, showed the same trend observed in steroid-dependent patients.

Regarding the relationship between endoleukocyte FA profile and trough MPA blood levels (Table 3), we found significant positive correlations for MPA and DHA (p-value 0.003) and MPA and n-3 (p-value 0.008) in SD patients. A negative correlation was observed for MPA and n-6 (p-value 0.008) and MPA and n-6/n-3 ratio (p-value 0.008). In contrast, no significant correlations were found between endoleukocyte FA profile and trough MPA blood levels in SR patients (Figures 1, 2). No significant correlations were found between tacrolimus trough levels and any component of the endoleukocyte FA profile.

**TABLE 3:** Correlations between trough blood MPA levels and FA profile of SD and SR patients.

​	​	​	​	​	n3	n6	n6/n3	20:4n6 (AA)	22:6n3 (DHA)
Spearman Rho	C0 MPA vs. SD patients	Coeff. correlation			0.72	−0.72	−0.72	0.22	0.76
		P-value			0.01	0.01	0.01	0.48	0.01
		n			12.00	12.00	12.00	12.00	12.00
		Bootstrap	Distorsion		−0.05	0.05	0.05	−0.01	−0.05
			Standard deviation		0.17	0.17	0.17	0.29	0.16
			95% conf. Int	Lower	0.23	−0.90	−0.90	−0.39	0.30
				Upper	0.90	−0.23	−0.23	0.74	0.96
	C0 MPA vs. SR patients	Coeff. correlation			0.14	−0.14	−0.14	−0.43	−0.09
		P-value			0.79	0.79	0.79	0.40	0.87
		n			6.00	6.00	6.00	6.00	6.00
		Bootstrap	Distorsion		−0.03	0.03	0.03	0.04	−0.01
			Standard deviation		0.55	0.55	0.55	0.44	0.47
			95% conf. Int	Lower	−1.00	−1.00	−1.00	−1.00	−1.00
				Upper	1.00	1.00	1.00	0.80	0.81
	C0 MPA vs. SR patients responding to CNI	Coeff. correlation			0.50	−0.50	−0.50	−0.50	0.50
		P-value			0.67	0.67	0.67	0.67	0.67
		n			3.00	3.00	3.00	3.00	3.00
		Bootstrap	Distorsion		−0.10	0.10	0.10	0.10	−0.10
			Standard deviation		0.81	0.81	0.81	0.81	0.81
			95% conf. Int	Lower	−1.00	−1.00	−1.00	−1.00	−1.00
				Upper	1.00	1.00	1.00	1.00	1.00

Correlations obtained with Spearman correlation analysis. MPA, micophenolic acid; AA, arachidonic acid; 20:4n6; DHA, docosaexaenoic acid 22:6n3; n-3, omega 3 PUFA; n-6, omega-6 PUFA. (−) indicates a negative correlation.

## Discussion

To the best of our knowledge, this is the first study describing an association between trough blood MPA levels and endoleukocyte FA profile in patients with INS.

Our results show that, in SDNS patients and in SRNS subjects in remission, trough MPA blood levels positively correlated with endoleukocyte anti-inflammatory n-3, while pro-inflammatory n-6 were negatively correlated. Moreover, in SDNS patients, trough blood MPA levels were positively correlated with DHA, the most important anti inflammatory fatty acid (Distefano et al., 2024; Kelley, 2001). In contrast, no correlation was observed in SRNS patients.

Our data also suggest that the immunosuppressive effect of MPA is expressed, among other mechanisms, through the activation of FA metabolism and underline a the possible role for achieving adequate trough blood MPA levels in patients with INS, in order to increase the anti-inflammatory n-3 activity, decrease the n-6 pro-inflammatory activity and counterbalance the inflammatory pathway of the disease (Harbige, 2003; Mayer et al., 2003). These MMF dose-dependent variations in n-3 and n-6 FA may also modulate the n-6/n-3 ratio in a way that promotes an anti-inflammatory environment within the lymphocyte cell.

On the other hand, the lack of correlation between tacrolimus trough levels and endoleukocyte FA profile in patients with SDNS may suggest that the above described associations were specific to MPA.

**FIGURE 3:**
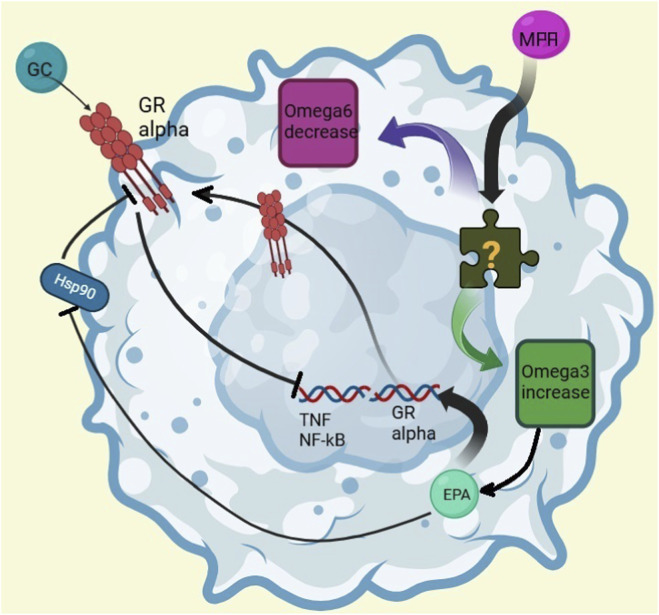
Putative mechanisms of interaction between MPA and fatty acids MPA: mycophenolic acid; GR: glucocorticoids receptor. GC, glucocorticoids. HSP90 protein: heat shock protein 90. EPA: eicosapentanoic acid. MPA increases omega-3 and decreases omega-6 levels in a dose-dependent manner, with a mechanism still unknown. Among the omega-3 series, EPA inhibits protein Hsp90 and enhances both the expression and activity of the GR alpha receptor. GR alpha, which is activated by glucocorticoids (GCs), suppresses the expression of inflammatory mediators such as Tumor Necrosis Factor (TNF) and Nuclear Factor-kB (NF-kB). However, Hsp90 counteracts this pathway by blocking GR alpha activity. (Figure made with Biorender on-line software, Toronto, Canada).

The different correlation between MPA levels and FA profile observed in SRNS patients deserves some comment. Of the six SRNS patients, three had persistent proteinuria, while the others were initially steroid resistant but eventually responded to immunosuppressive drugs and achieved remission. It is interesting to note that the three SR patients in remission showed the same, although not statistically significant, trend, while the three with persistent proteinuria did not. We can first hypothesize that proteinuria itself explains the lack of correlation between MPA and FA in this last cohort. There is no evidence in the literature that proteinuria modifies leukocyte biology, while it is known that it can alter FA metabolism (Soria-Castro et al., 2020). Another possible mechanism explaining the above finding involves the expression of chaperonine Hsp90 and glucocorticoid receptors (GR). It is known that the ratio of chaperonine Hsp90 and GR is considered a factor of steroid resistance (Tissing et al., 2005). It has been described that chaperonine Hsp90 urinary levels are elevated during proteinuria and prednisolone is able to decrease its levels (Aks et al., 2024). In addition, chaperonine Hsp90 expression in the GR complex is decreased and GR expression is increased (Wu et al., 2021) by n-3 EPA (eicosapentanoic acid, 20:5n3), resulting in increased responsiveness to glucocorticoids (Wu et al., 2021). We can thus hypothesize that the lack of n-3 increase, observed in the three patients with multi drug-resistant NS, could represent one of the mechanisms (Figure 3) behind the steroid resistance of these patients. Unfortunately, our data do not allow us to explore this mechanism in depth, but rather to envision a path to take in future research.

A strong point of our study is the precise characterization of the patients, including the availability of their diet, which is a relevant environmental variable influencing blood FA profile. The second strong point consists in the study of FA profile inside the leukocytes, which may provide more specific information on the activity of the immune system, in the course of nephrotic syndrome, than the study in the blood, influenced by various metabolic factors resulting from proteinuria (Turolo et al., 2025).

On the other hand, a weakness of our study is the limited cohort size, as it was designed as a first exploratory single-center pilot study, analysis was conducted only on MPA trough blood level and not also on the drugs’s area under the curve (AUC), due the lack of this data.

Our findings indicate that therapeutic MPA trough levels are associated with a shift in the PUFA pathway toward a more favorable intracellular n-6/n-3 ratio and increased DHA levels, likely supporting the natural anti-inflammatory activities of n-3 fatty acids. Based on this, the present study may reinforce the need to achieve adequate MPA trough levels during the course of SDNS. In SRNS with persistent proteinuria, no enhancement of omega-3–mediated anti-inflammatory activity was observed despite therapeutic MPA levels, thereby opening the way for further studies on fatty acids as potential intermediate mechanisms of steroid resistance.

Our work fits within the broader research framework focused on optimizing drug use and nutritional approach to improve outcomes in children with INS. To this end, we have recently published (Morello et al., 2025) that MPA trough levels greater than or equal to 3 ng/mL were associated with longer periods of remission compared with those lower than 3.

In conclusion, our results support the clinical value of therapeutic drug monitoring of MPA. They also contribute to the understanding the biological mechanisms that influence inflammatory responses and eventually the course of the disease. This perspective strengthens the importance of an integrated pharmacological and dietary approach in children with NS, which is also potentially applicable to other diseases, such as Systemic Lupus Erythematosus, or to transplanted patients, settings in which MMF is commonly used. Moreover, it will be useful to explore the correlation of endoleukocyte n-3 and n-6 to MPA AUC.

## Data Availability

The original contributions presented in the study are included in the article/supplementary material, further inquiries can be directed to the corresponding author.
